# Increased phosphorylation of Cx36 gap junctions in the AII amacrine cells of RD retina

**DOI:** 10.3389/fncel.2015.00390

**Published:** 2015-10-01

**Authors:** Elena Ivanova, Christopher W. Yee, Botir T. Sagdullaev

**Affiliations:** Departments of Ophthalmology and Neurology, Burke Medical Research Institute, Weill Medical College of Cornell UniversityWhite Plains, NY, USA

**Keywords:** retinal degeneration, oscillations, AII amacrine cells, gap junctions, phosphorylation, hyperactivity, spontaneous activity

## Abstract

Retinal degeneration (RD) encompasses a family of diseases that lead to photoreceptor death and visual impairment. Visual decline due to photoreceptor cell loss is further compromised by emerging spontaneous hyperactivity in inner retinal cells. This aberrant activity acts as a barrier to signals from the remaining photoreceptors, hindering therapeutic strategies to restore light sensitivity in RD. Gap junctions, particularly those expressed in AII amacrine cells, have been shown to be integral to the generation of aberrant activity. It is unclear whether gap junction expression and coupling are altered in RD. To test this, we evaluated the expression and phosphorylation state of connexin36 (Cx36), the gap junction subunit predominantly expressed in AII amacrine cells, in two mouse models of RD, rd10 (slow degeneration) and rd1 (fast degeneration). Using Ser293-P antibody, which recognizes a phosphorylated form of connexin36, we found that phosphorylation of connexin36 in both slow and fast RD models was significantly greater than in wildtype controls. This elevated phosphorylation may underlie the increased gap junction coupling of AII amacrine cells exhibited by RD retina.

## Introduction

Retinal degeneration (RD) encompasses a family of diseases originating from genetic alteration in more than 100 genes, as well as acquired conditions, such as trauma caused by excessive light exposure, and aging (Menzler et al., 2014). While the most apparent physiological consequence of RD is the progressive loss of photoreceptors, surviving inner retinal neurons form a dysfunctional network that generates aberrant synaptic activity (Pu et al., 2006; Marc et al., 2007; Stasheff, 2008). This aberrant activity acts as a barrier to signal transmission within the retina (Yee et al., 2012). Similar rhythmic activity has been observed in higher visual centers in RD models (Drager and Hubel, 1978; Sauve et al., 2001; Ivanova et al., 2015) and may be the cause of flashing sensations reported by RD patients (Lepore, 1990; Murtha and Stasheff, 2003). These studies suggest that the degradation of signal fidelity in the retina may translate to visual impairment. A finding showing that reducing this aberrant activity can restore visual responses in degenerated retina provides new insights into our understanding of degenerative process and opens up a new venue for therapy (Toychiev et al., 2013a).

A search for the source of aberrant activity in RD has led to the oscillatory behavior of the AII amacrine cell, a specialized interneuron at the crossroads of rod and cone photoreceptor signaling pathways in the inner retina (Trenholm et al., 2012; Choi et al., 2014). AII amacrine cell oscillations are shown to be reliant on interactions via open gap junctions. Experiments using the gap junction permeable tracer Neurobiotin revealed an increase in labeling spread, indicative of elevated coupling of AII amacrine cells (Ivanova et al., 2015). Accordingly, blocking gap junctions eliminates aberrant activity in RD and restores sensitivity to light stimuli (Toychiev et al., 2013a). The genetic elimination of connexin36, a subunit prevalent in gap junctions formed by AII amacrine cells, has a similar effect in RD mice (Ivanova et al., 2015).

Oscillatory activity of AII amacrine cells, however, is not unique to RD; AII amacrine cells can be brought to oscillate in wt retina (Cembrowski et al., 2012). This suggests that, in RD, upstream retinal processes are modified in a way that perturbs AII cell homeostasis, leading to increased coupling and membrane oscillations. While the role of coupled AII amacrine cells is becoming clear, the changes in gap junctions that lead to aberrant activity are unknown. In healthy tissue, gap junction coupling is modulated through phosphorylation at serine 293 (Ser293; Kothmann et al., 2009, 2012; Meyer et al., 2014). It is unknown, however, whether the same functional mechanism is involved in RD, or whether it is due to structural changes causing increased gap junction expression.

To test this hypothesis, we evaluated the expression patterns of connexin36-positive plaques (structural assay) and state of connexin36 phosphorylation (functional assay) in AII amacrine cells in two mouse models of RD (rd10, late onset and slow degeneration; rd1, early onset and fast degeneration) using specific antibodies to phosphorylated form of connexin36 (a gift of John O’Brien, from Kothmann et al., 2009, 2012). We found in AII amacrine cells that the density and size of the connexin36-positive plaques, as well as the amount of connexin36 in a single plaque, were similar between wt and both slow and fast RD mouse models. Strikingly, connexin36 was highly phosphorylated in AII amacrine cells in both rd10 and rd1 mice, compared to wt. This elevated phosphorylation suggests that AII amacrine cells, normally uncoupled during light adaptation in wt, remained coupled in RD retinas.

## Materials and Methods

### Animals

In all experimental procedures, animals were treated according to regulations in the ARVO Statement for the Use of Animals in Ophthalmic and Vision Research, in accordance with protocols approved by the Institutional Animal Care and Use Committee of Weill Cornell Medical College, and the NIH Guide for the Care and Use of Laboratory Animals. Wildtype (C57BL/6J; RRID:IMSR_JAX:000664), rd10 (B6.CXB1-Pde6brd10/J; RRID:IMSR_JAX:004297), and rd1 (B6.C3-Pde6brd1 Hps4le/J RRID:IMSR_JAX:000659) mice of either sex were obtained from the Jackson Laboratory (Bar Harbor, ME) and aged to 8–9 months.

### Viral Injections

AII amacrine cells were visualized by injection of a recombinant adeno-associated virus serotype 2 (rAAV2) carrying a construct of green fluorescent protein (GFP) under control of a cytomegalovirus (CMV) promoter as previously described (Ivanova and Pan, 2009). The rAAV2 carries a Y444F capsid mutation for highly efficient vector transduction (Petrs-Silva et al., 2009). Briefly, mice aged postnatal day 30–60 (P30–60) were anesthetized by intraperitoneal injection of a mixture of 150 mg/kg ketamine and 15 mg/kg xylazine. Under a dissecting microscope, a small perforation was made in the temporal sclera region with a sharp needle. A total of 1.5 μl viral vector suspension in saline was injected into the intravitreal space through the perforation with a glass pipette (1B150F-4; WPI, Sarasota, FL, USA) pulled with a P-97 Flaming/Brown puller (Sutter Instruments, Novato, CA, USA). Viral vectors were packaged and affinity purified by Virovek (Hayward, CA, USA).

### Immunohistochemistry

#### Preparation of Retinal Whole Mounts

Eight to nine month old light-adapted mice were euthanized at the same time of the day (12 pm). As previously described (Ivanova et al., 2013; Toychiev et al., 2013b), the eyes were enucleated and placed in bicarbonate-buffered Ames’ medium equilibrated with 95% O_2_ and 5% CO_2_. The cornea was removed by the encircling cut above *ora serrata*. The iris, lens, and vitreous were removed. The remaining eyecup, with the retina still attached to the pigment epithelium, was cut into two halves. This preparation was transferred to a small petri dish and incubated for 40 min at 35°C in Ames’ medium equilibrated with 95% O_2_ and 5% CO_2_ under bright light.

The eyecups with the attached retinas were submersion-fixed in freshly prepared 4% carbodiimide in 0.1 M phosphate saline (PBS, pH = 7.3) for 15 min at room temperature. After fixation, the eyecups were washed in PBS and the retinas were separated from the eyecups. Retinal wholemounts were blocked for 10 h in a PBS solution containing 5% Chemiblocker (membrane-blocking agent, Chemicon), 0.5% Triton X-100, and 0.05% sodium azide (Sigma). Primary antibodies were diluted in the same solution and applied for 72 h, followed by incubation for 48 h in the appropriate secondary antibody, conjugated to Alexa 488 (1:800; green fluorescence, Molecular Probes), Alexa 568 (1:800; red fluorescence, Molecular Probes), Alexa 647 (1:100; far red fluorescence, Molecular probes), or Cy5 (1:400; far red fluorescence, Jackson ImmunoResearch). In multi-labeling experiments, wholemounts were incubated in a mixture of primary antibodies, followed by a mixture of secondary antibodies. All steps were carried out at room temperature. After staining, the retina was flat mounted on a slide, ganglion cell layer up, and coverslipped using Vectashield mounting medium (H-1000, Vector Laboratories). The coverslip was sealed in place with nail polish. To avoid extensive squeezing and damage to the retina, small pieces of a broken glass cover slip (Number 1 size) were placed in space between the slide and the coverslip. The primary antibodies used in this study were the following: mouse anti-Cx36 (mCx36, 1:1000, EMD Millipore Cat# MAB3045 RRID:AB_94632), rabbit anti-Ser293-P (Ser293-P, 1:3000, kind gift from Dr. J. O’Brien, The University of Texas; Kothmann et al., 2007).

Monoclonal mCx36 was originally produced in perch as an antibody to Cx35 (which is homologous to mammalian Cx36) and verified by Western blot (Pereda et al., 2003; O’Brien et al., 2004). The density of Cx36-immunoreactive plaques in our wt mice (202 ± 7 plaques per 10^3^ μm^2^) which is similar to what has been found previously in mouse retina (272–297 plaques per 10^3^ μm^2^; Meyer et al., 2014). A significantly higher density was found in rabbit (~40 plaques per 10^3^ μm^2^; Kothmann et al., 2009). The average size of our plaques (0.25 ± 0.03 mm^2^) was again similar to what was previously reported (0.2 mm^2^), in a study that also found the majority of Cx36 gap junctions to occur between AII amacrine cells (homocellular coupling), with a minority (~30 out of 170 clusters) occurring between AII amacrine cells and ON cone bipolar cells (heterocellular coupling; Meyer et al., 2014). Again, Cx36 were larger in rabbit (~0.75 mm^2^; Kothmann et al., 2009) than in mouse. Ser293-P antibody was originally produced to recognize the phosphorylated form of Cx35 in perch (Kothmann et al., 2007). Since Cx36 is the mammalian homolog of Cx35, the Ser293-P antibody also recognizes the mammalian Cx36 protein (rabbit, Kothmann et al., 2009, 2012; mouse, Li et al., 2013), and Western blot showed that the antibody was specific for the phosphorylated form of the protein, and did not cross-react with the other phosphorylation sites.

#### Preparation of Retinal Cryostat Sections

After fixation, the eycups were washed in PBS and cryoprotected in a sucrose gradient (10, 20 and 30% w/v in PBS). The retina was separated from the eyecup and frozen in OCT medium. Cryostat sections were cut at a thickness of 20 microns and attached to microscope slides. The immunolabeling was performed as for the retinal wholemounts except the incubations steps were shortened as the following: blocking for 1 h, incubation with a mixture of the primary antibodies for 12 h, incubation with a mixture of the secondary antibodies for 1 h. The antibody dilutions were the same as for wholemounts.

### Data Acquisition and Analysis

Retinal wholemounts were evaluated under Nikon Eclipse Ti-U confocal microscope using with a x60 oil objective (1.4 N.A.). The retinas from all mouse lines were imaged under identical acquisition conditions, including: laser intensity, photomultiplier amplification, and *z*-stack step size. Three animals from each mouse line were examined. The analysis was conducted as described earlier (Kothmann et al., 2009, 2012). First, the mCx36 labeling was set at fixed threshold level. Second, each connexin36-positive plaque, immunolabeled by mCx36, was identified using particle recognition algorithm. In total for each type of the analyses, we evaluated 15318 individual plaques in wt, 13171 plaques in rd10, and 15907 plaques in rd1. Each mouse line was represented by three different animals; each retina was analyzed in four 78.5 × 78.5 mm^2^ fields.

All images were processed and were analyzed using ImageJ software (ImageJ, RRID:nif-0000-30467). The resulting data are presented as mean ± standard error. Unless otherwise specified, statistical analyses were performed using a one-way Analysis of Variance (ANOVA).

## Results

### Specificity of Anti-Connexin36 Antibodies

**Figure 1:**
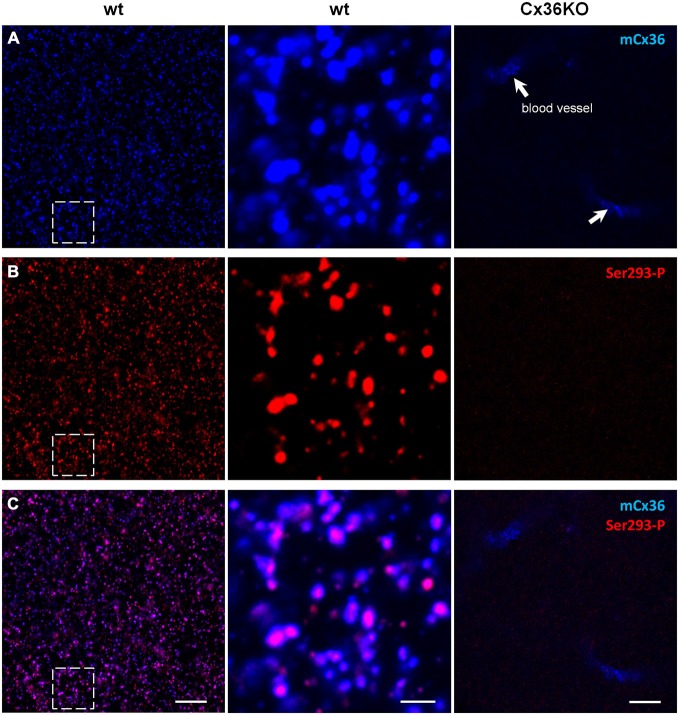
**Ser293-P antibody specifically recognizes Cx36 in the mouse retinal wholemounts.** Confocal images show the lamina of the inner plexiform layer (IPL) adjacent to the retinal ganglion cell bodies. **(A)** mCx36 (blue) staining in wt retina (left). Magnified region shows strong punctate labeling (middle). No punctate labeling was present in Cx36KO retina, though there was some non-specific labeling of blood vessels by secondary anti-mouse antibodies (arrows). Their stereotypic localization of vascular elements in the IPL was used as a marker for specific retinal strata. The brightness of mCx36 puncta was increased for presentation purposes only to reveal all Cx36-positive plaques. **(B)** In the same region of wt retina as in **(A)**, Ser293-P staining was present in wt retina, with similar punctate labeling. No Ser293-P staining was present in Cx36KO retina. The degree of phosphorylation, reflected by the intensity of the Ser293-P labeling, varied across Cx36 plaques. **(C)** The magnified region (middle) shows that the vast majority of Ser293-P-positive puncta colocalized with mCx36-postive puncta in wt retina. 97.8 ± 0.3% were colocalized with mCx36 (3 retinas × 3 samples = 976 detectably phosphorylated plaques). Scale bars: 10 μm, left and right columns; 2 μm, middle column.

Connexin36 (Cx36) is a gap junction subunit found predominantly in AII amacrine cells, and thought to be essential for aberrant activity in RD (Trenholm et al., 2012; Choi et al., 2014; Ivanova et al., 2015). First, we tested antibodies to detect connexin36 gap junctions. Monoclonal mCx36 antibody was used to identify connexin36 (Pereda et al., 2003; O’Brien et al., 2004). Phosphorylation of connexin36 at the Ser293 site increases gap junction coupling (Kothmann et al., 2009). Ser293-P antibody (a gift from Dr. John O’Brien) was used to visualize phosphorylated connexin36 and thus to identify coupled gap junctions. This antibody has been previously tested in perch (Kothmann et al., 2007) and rabbit (Kothmann et al., 2009, 2012) retinas, as well as the outer plexiform layer of mouse retina (Li et al., 2013). Here, we will focus on Ser293-P expression in the mouse inner plexiform layer (IPL), which contains the processes of AII amacrine cells. We double labeled connexin36 knockout (Cx36KO) mice (as a negative control) and wildtype C57BL/6 mice with antibodies for mCx36 and Ser293-P (Figure 1). Neither antibody showed punctate staining in Cx36KO retina (Figure 1A). There was some expected non-specific labeling of blood vessels produced by the secondary anti-mouse antibody but not by the primary mCx36 antibody. This was tested by omitting the primary mCx36 antibodies from the staining procedure (data not shown), which is consistent with previous work that has shown that secondary anti-mouse antibodies can be used to label retinal blood vessels in mouse (Ivanova et al., 2014).

In the IPL of wt, mCx36 recognized multiple connexin36 plaques (Figure 1A). In Cx36KO retina, the characteristic for wt punctate labeling disappeared consistent with the high specificity of this antibody to connexin36 protein (Figure 1A). Among Ser293-P-positive puncta, 97.8 ± 0.3% were colocalized with mCx36-labeled plaques in wt retina (3 retinas × 3 samples = 976 detectably phosphorylated plaques), which shows that this antibody are highly specific for connexin36. Consistently, the staining for Ser293-P was completely abolished in Cx36KO mouse. The variations in intensity of individual Ser293-P-positive plaques in wt mouse most likely reflect the differences in the amount of phosphorylation of connexin36. In summary, using the retinas of wt and Cx36KO mice we confirmed high specificity of mCx36 and Ser293-P antibodies for connexin36 protein.

### Connexin36 Localization in AII Amacrine Cells

**Figure 2:**
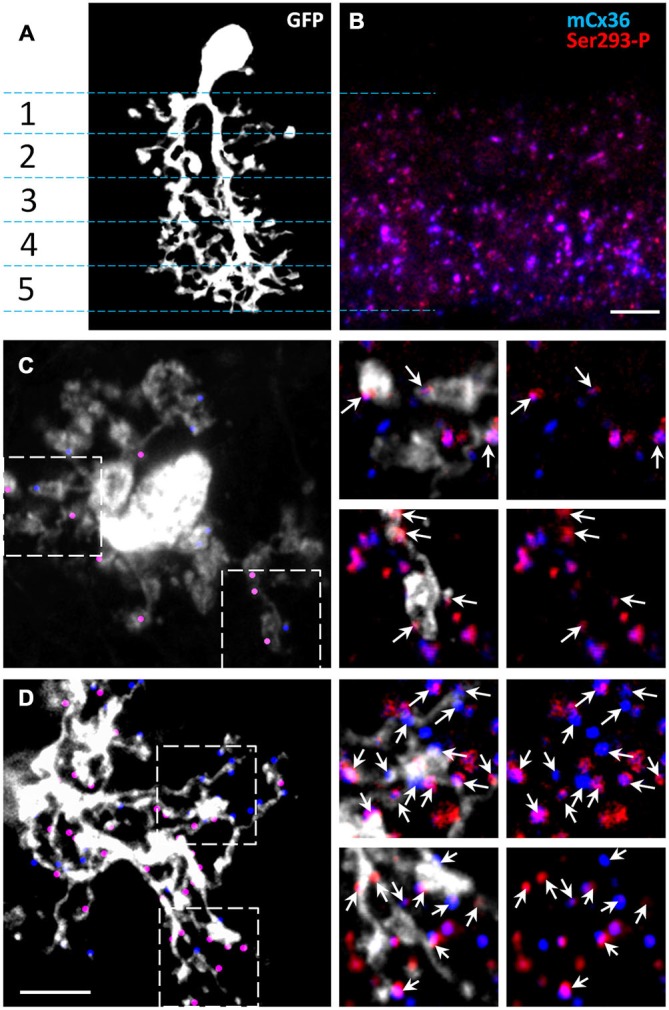
**The majority of Cx36 gap junctions in AII amacrine cells are on the arboreal dendrites. (A)** Confocal *z*-stack reconstruction of AII amacrine cell in retina wholemount selectively labeled by recombinant adeno-associated virus serotype 2 (rAAV2)-green fluorescent protein (GFP). In the vertical retinal view, stratification of a single AII amacrine cell (white) is shown through the five IPL strata. **(B)** In the 1.5 um thick vertical cryostat section, matched to the IPL area in **(A)**, Cx36 puncta were predominantly present in the On-sublamina (strata 3–5). Cx36 were indirectly labeled by the mCx36 antibody (blue) and phosphorylated Cx36 at Ser293 were recognized by Ser293-P antibody (red). **(C,D)** In a retinal wholemount, a rAAV2-GFP-labeled AII amacrine cells expressed more Cx36 in stratum 5 (**D**, marked by dots) than in stratum 2 **(C)**. Single confocal sections from the boxed areas were magnified (×6.2) and are shown on the right. Among all Cx36-positive puncta, the puncta colocalized with the processes of the AII amacrine cell are marked by arrows. Within the processes of the same AII amacrine cell different amount of phosphorylation was detected. Scale bars: 5 μm.

The dendrites of AII amacrine cells span the entire IPL, which can be subdivided into five strata (Ghosh et al., 2004). To investigate the phosphorylation state of connexin36 in AII amacrine cells, we first determined where connexin36 puncta colocalized with AII amacrine cell processes in wt retina (Figure 2A). Isolated AII amacrine cells were imaged from wholemount preparations and reconstructed from a *z*-stack of confocal images. AII amacrine cells were visualized by GFP expression conferred to the cells via the viral transfer. As previously shown, after viral intraocular injections, GFP expression in the inner nuclear layer was predominantly localized to AII amacrine cells (Ivanova and Pan, 2009). The majority of connexin36-positive puncta were found in strata 3–5, which comprise the ON sublamina of the IPL, whereas strata 1–2, which comprise the OFF sublamina, had relatively sparse connexin36 expression (Figure 2B). Next, we tested whether connexin36 puncta colocalized with the lobular appendages (stratum 2) and arboreal dendrites (stratum 5). Connexin36-positive puncta were rarely colocalized with lobular appendages (Figure 2C), while they were prevalent along arboreal dendrites (Figure 2D).

Our data in mice are consistent with and extend the findings of previous studies conducted in rabbits and rats (Feigenspan et al., 2001; Mills et al., 2001; Kothmann et al., 2009). Similar to our data in mouse, little connexin36 colocalization was found at the lobular appendages and somas of AII amacrine cells in the OFF sublamina. Instead, connexin36 puncta appeared to be mostly restricted to the ON sublamina of the IPL, where in stratum 5 96–98% of puncta were located on the arboreal dendrites of AII amacrine cells (Feigenspan et al., 2001; Mills et al., 2001). Thus, in stratum 5, the majority connexin36 puncta colocalized with AII cell processes. Therefore, in the next set of experiments, we evaluated the distribution and phosphorylation state of connexin36 in AII amacrine cells within stratum 5 of the IPL.

### Connexin36 Phosphorylation is Elevated in RD

**Figure 3:**
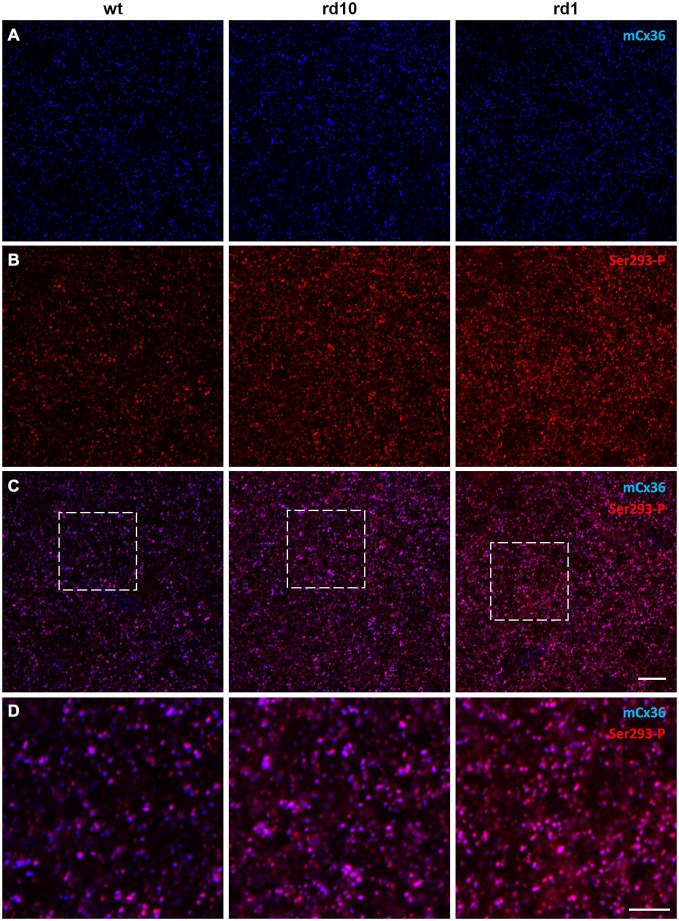
**Phosphorylation of Cx36 gap junctions is elevated in RD retina. (A–C)** Single confocal sections through the stratum 5 of the retinal IPL labeled for Cx36 (mCx36, blue) and its phosphorylated form (Ser293-P, red) are shown for wt (left column), rd10 (middle), and rd1 (right). **(D)** The magnified boxed areas from the merged images are shown in the bottom row. High amount of phosphorylation, reflected by pink color in the merged images, was characteristic for both rd10 and rd1 retinas. Scale bars: 10 μm.

To determine how connexin36 expression and phosphorylation in AII amacrine cells is altered in RD, we compared light-adapted retinas of wt mice to two models of RD: rd10 (slow degeneration) and rd1 (fast degeneration). All retinal wholemounts, including wt controls, were processed in parallel under identical conditions and double labeled with antibodies for mCx36 and Ser293-P to label connexin36 and phosphorylated connexin36, respectively (Figure 3). The density and distribution of mCx36 (blue) was similar in the three mouse lines (Figure 3A). However, the density and intensity of the labeling for the phosphorylated form of connexin36 (red) were higher in both RD models relative to wt (Figure 3B). In the merged images of mCx36 and Ser293-P, more overlap (pink) was present in the retinas of RD mice, suggesting that more puncta were double labeled in RD (Figure 3C). Consistently, in the magnified boxed areas (Figure 3D), the majority of connexin36 puncta were phosphorylated (pink) in rd10 and rd1, in contrast to a greater amount of non-phosphorylated (blue) puncta in wt. The phosphorylation state of connexin36 was very heterogeneous in wt, while it was more even and consistently elevated in RD retinas.

**Figure 4:**
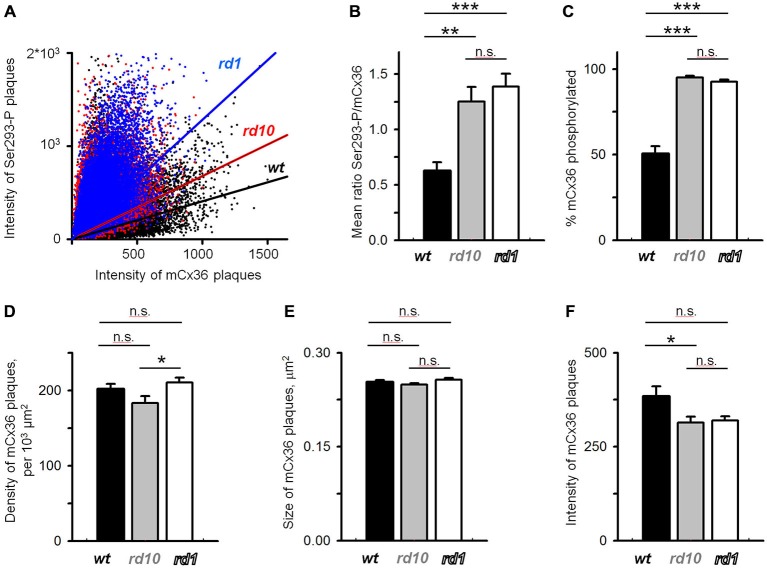
**Quantification of Cx36 gap junctions in AII amacrine cells in wt, rd10 and rd1 retinas. (A)** Cx36 was heterogeneously phosphorylated in the retinas of all mouse models, which is reflected by a low correlation between the intensity of Cx36 and its phosphorylation (wt *r*^2^ = 0.096; rd10 *r*^2^ = 0.131; rd1 *r*^2^ = 0.299). **(B)** Relative phosphorylation of Cx36, extracted from the data in **(A)**, shows overall more phosphorylation in retina of RD models. **(C)** The absolute amount of phosphorylation, reflected by the percentage of Cx36 plaques that show detectable Ser293-P labeling, was also elevated in RD retinas. **(D,E)** The density of Cx36-positive plaques **(D)**, their size **(E)**, and intensity of mCx36 labeling **(F)** were not significantly altered in RD models. The data are presented as average; error bars are SEM. All significances are based on ANOVA test, where ***p < 0.001, **0.001 < *p* < 0.01, *0.01 < *p* < 0.05, n.s. *p* > 0.05.

To quantify these changes, mCx36 and Ser293-P immunofluorescence were analyzed within individual regions of interest defined by threshold detection algorithm (see “Materials and Methods” Section). These connexin36-positive puncta are hereafter referred to as connexin36 plaques. In this analysis, we assumed that all connexins in a single plaque were labeled by mCx36 and thus, the intensity of the immunolabeling reflected the number of connexins. The individual plaques of connexin36 existed in different phosphorylation states (Figure 4A). This was most apparent in wt retinas, where there was little or no correlation between the intensity of mCx36 labeling and the intensity of Ser293-P (wt *r*^2^ = 0.096). In contrast to wt, the amount of phosphorylation in RD mouse lines correlated with the general amount of connexin36 in a plaque (rd10 *r*^2^ = 0.131; rd1 *r*^2^ = 0.299). This is due to overall high phosphorylation level of connexin36 in rd10 and especially rd1. Next, to determine the relative amount of phosphorylation in a plaque, we calculated the ratio of Ser293 phosphorylation to the total amount of connexin36 (Kothmann et al., 2009). The ratio was significantly higher in rd10 (1.25 ± 0.13, *n* = 12 retinal segments, *p* = 0.0012) and rd1 (1.38 ± 0.12, *n* = 12, *p* < 0.0001) retinas in comparison to wt (0.62 ± 0.08, *n* = 12; Figure 4B). The difference was even more pronounced in the number of detectably phosphorylated connexin36 (Figure 4C). In wt, around 50% of connexin36 plaques were phosphorylated (50 ± 4%); whereas, in RD retinas, more than 90% of the plaque had detectable phosphorylation (rd10 95 ± 1%; rd1 92 ± 1%, *p* < 0.0001 for both).

### Connexin36 Expression is not Altered in RD

The increased levels of connexin36 phosphorylation in rd10 and rd1 retinas indicates that functional state of gap junctions is altered in RD. Next, we wanted to determine whether the expression level of connexin36 was affected in RD. Here, we analyzed the density of connexin36-positive plaques, their size and the intensity of the individual plaques, a common metric for the number of connexin36 within the plaque. We found no significant difference in density between wt (202 ± 7 per 10^3^ μm^2^, *n* = 11) and rd10 (183 ± 9 per 10^3^ μm^2^, *n* = 12, *p* = 0.22) or rd1 (211 ± 6 per 10^3^ μm^2^, *n* = 12, *p* = 0.71; Figure 4D). Density was slightly elevated in rd1 than rd10 (*p* = 0.049). This lack of pronounced changes in density of connexin36 plaques in RD is intriguing and may be associated with apparent anatomical changes in AII amacrine cells during progression of RD (Barhoum et al., 2008). There was no difference in the size of mCx36 plaques across genotypes (wt 0.25 ± 0.04 μm^2^; rd10 0.25 ± 0.03 μm^2^; rd1 0.26 ± 0.03 μm^2^; *p* > 0.51 for each comparison; Figure 4E). The intensity of mCx36 labeling in wt retina (383 ± 27) was slightly higher than rd10 (314 ± 16; *n* = 12, *p* = 0.047) and non-significantly higher than rd1 (319 ± 11; *n* = 12, *p* = 0.071). In total for each panel of Figure 4, we evaluated 15,318 individual plaques in wt, 13,171 plaques in rd10, and 15,907 plaques in rd1. Each mouse line was represented by three different animals; each retina was analyzed in four 78.5 × 78.5 mm^2^ fields.

In conclusion, these data suggest the level of connexin36 phosphorylation but not the density or the size of connexin36 plaques were changed in AII amacrine cells in RD retinas.

## Discussion

The major finding of this study was that connexin36 gap junctions in AII amacrine cells exhibit elevated phosphorylation in both fast (rd1) and slow (rd10) mouse models of RD, while the general expression level of connexin36 remained unchanged. This elevated phosphorylation may underlie the increased gap junction coupling of AII amacrine cells exhibited by RD retina (Ivanova et al., 2015).

### Gap Junctions Coupling and Hyperactivity

The importance of gap junctions for hyperactivity in AII amacrine cells in RD has been established (Trenholm et al., 2012; Choi et al., 2014); however, the precise mechanism by which gap junctions promote oscillations is unknown. Does this mechanism require structural changes and anatomical rewiring well documented in RD retina (Strettoi and Pignatelli, 2000; Strettoi et al., 2002; Marc et al., 2007; Chua et al., 2009; Puthussery et al., 2009; Phillips et al., 2010; Haq et al., 2014) or would the alteration of normal retinal physiology be sufficient?

In a healthy retina, electrical synapses formed by gap junctions between neurons underlie a number of important functions, including transmission of rod-driven signals (Deans et al., 2002), neuronal synchronization (Deans et al., 2001), signal averaging (DeVries et al., 2002), and signal gain control (Vardi and Smith, 1996; Dunn et al., 2006). Changes in light levels affect the coupling state of gap junctions, which affects how cells, particularly groups of cells, process visual signals. What happens with the gap junctions when the light-sensitive cells, the photoreceptors, die in RD? In our hands, the most striking difference between wt and RD retinas was the elevated phosphorylation of connexin36 in AII amacrine cells. The phosphorylation was shown to be associated with the coupling among AII amacrine cells (Kothmann et al., 2009, 2012). These findings indicate that, in contrast to wt, AII amacrine cells are coupled in light-adapted RD retinas. Physiologically, the coupling of AII amacrine cells was shown to be essential for generation and/or propagation of the aberrant hyperactivity (Trenholm et al., 2012). Is this coupling unique for RD retinas? The following line of evidence suggests that both coupling of AII amacrine cells and the aberrant activity could be induced in wt retina. First, in wt retina, the phosphorylation could be adjusted to homogeneously high level with pharmacological agents (Kothmann et al., 2009, 2012). Second, gap junctions in wt retina are naturally coupled or uncoupled depending on the light adaptation state of the retina (Hampson et al., 1992; Witkovsky, 2004). Finally, AII amacrine cells can be brought to oscillate in wt retina (Cembrowski et al., 2012). Moreover, deprivation of the healthy wt retina of photoreceptor input via pharmacological agents or bleaching could reproduce the oscillations in AII amacrine cells with the same pharmacological profile and frequencies as seen in RD (Trenholm et al., 2012; Menzler et al., 2014). These oscillations appear to be a property of a gap junction coupled network and disappear when gap junctions are blocked (Trenholm et al., 2012; Margolis et al., 2014). Thus, our research is in line with reports from other groups that suggest that both elevated coupling and aberrant activity are not unique for RD. Instead, the endogenous regulation of gap junctions in AII amacrine cells could be compromised in RD, leading to their extensive coupling and the aberrant activity.

### Regulation of Connexin36 Gap Junctions in AII Amacrine Cells in Health and Disease

**Figure 5:**
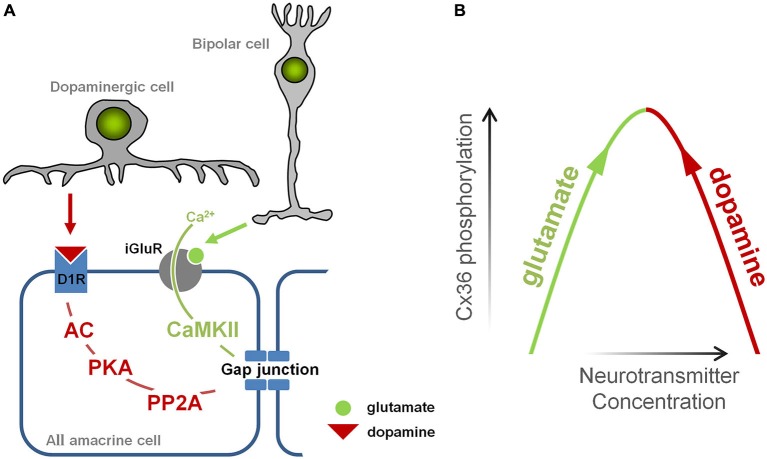
**Illustration of the events leading to opening and closing of gap junctions in AII amacrine cell. (A)** Glutamate, released from bipolar cells, induces Ca^2+^ influx via N-methyl-D-aspartate (NMDA) receptors which in turn mediates CaMKII-dependent phosphorylation of Cx36 and opens gap junctions. Dopamine, released by dopaminergic amacrine cells, binds to D1-like receptors activating phosphatase protein phosphatase 2A (PP2A) which in turn dephosphorylates Cx36 and closes gap junction. **(B)** The diagram illustrating a mechanism by which changes in glutamate and dopaminergic signaling may lead to increased phosphorylation of Cx36-containing gap junctions promoting coupling of AII amacrine cells.

In healthy retina, gap junction coupling in AIIs is tightly controlled by two main neurotransmitters: glutamate and dopamine, which are released onto AII amacrine cells by bipolar cells and dopaminergic amacrine cells, respectively (Figure 5A). Glutamate leads to the activation of calmodulin kinase II (CaMKII), which phosphorylates Cx36, promoting gap junction coupling (Kothmann et al., 2012); dopamine leads to the activation of protein kinase alpha (PKA), which activates another enzyme, protein phosphatase 2A (PP2A) that dephosphorylates Cx36, reducing gap junction coupling (Kothmann et al., 2009). Thus, gap junctions in AII cells are controlled by two pathways that normally act in *opposing* fashion: the glutamatergic pathway opens gap junctions, while the dopaminergic pathway closes them.

In RD, there is evidence that both pathways are altered. Increased glutamate signaling develops during RD, and originates from the increased spontaneous activity of bipolar cells (Marc et al., 2007; Borowska et al., 2011). Meanwhile, dopaminergic amacrine cells, the only source of dopamine in the retina, have dramatically reduced bursting activity in RD (Atkinson et al., 2013), which parallels changes in dopamine metabolism, including reduced levels of dopamine (Djamgoz et al., 1997) and its metabolites DOPA and DOPAC (Nir and Iuvone, 1994; Nir et al., 2000; Park et al., 2013). In other words, the aberrant activity of bipolar cells increases glutamatergic input to amacrine cells, encouraging gap junction opening, while the diminished output of dopaminergic amacrine cells decreases dopaminergic input to amacrine cells, discouraging gap junction closing. Therefore, in RD, the glutamatergic and dopaminergic pathways are altered in a way that they act *synergistically* to open gap junctions (Figure 5B).

In conclusion, our work expands the understanding of gap junction coupling, and outlines new potential targets for the elimination of aberrant activity and restoration of light responses in RD. It is important to note, however, that gap junction coupling is also elevated in mesopic lighting conditions (Bloomfield and Volgyi, 2004). Therefore, aberrant activity in RD cannot be attributed to this single intrinsic mechanism alone. Future studies are needed to distinguish the contribution of altered normal physiology and maladaptive changes that occur as RD progresses and to further elucidate the complex etiology and nature of physiological remodeling in RD.

## Conflict of Interest Statement

The authors declare that the research was conducted in the absence of any commercial or financial relationships that could be construed as a potential conflict of interest.
